# Pretreatment with an anti-CGRP monoclonal antibody attenuates mild TBI-induced tactile hypersensitivity in mice

**DOI:** 10.1186/s10194-025-02108-x

**Published:** 2025-08-04

**Authors:** Anne-Sophie Wattiez, Adisa Kuburas, William C. Castonguay, Kim Fejgin, Ib V. Klewe, Andrew F. Russo

**Affiliations:** 1 Department of Molecular Physiology and Biophysics, University of Iowa, 51 Newton Rd., Iowa City, IA 52242 USA; 2 Department of Neurology, University of Iowa, Iowa City, IA 52242 USA; 3 Center for the Prevention and Treatment of Visual Loss, Veterans Administration Health Center, Iowa City, IA 52246 USA; 4 H. Lundbeck A/S, Research DK, Valby, Denmark; 5 Present Address: Center for Scientific Review, National Institutes of Health, Bethesda, MD 20982, USA; 6 Present Address: Department of Neurology Translational Research, University of Utah Hospitals and Clinics, Salt Lake City, UT 84132 USA

**Keywords:** Traumatic brain injury, Post-traumatic headache, Calcitonin gene-related peptide, Monoclonal antibody

## Abstract

**Background:**

Post-traumatic headache (PTH) can develop following a mild traumatic brain injury (mTBI), such as a concussion. It is especially common among active-duty military personnel, Veterans, and athletes. The high prevalence and chronic nature of PTH highlight the importance of studying these conditions in animal models to develop new, effective treatments. Since the symptoms associated with PTH resemble those of migraine, we focused on the neuropeptide calcitonin gene-related peptide (CGRP) as a potential therapeutic target.

**Methods:**

We used a mouse model of mTBI involving three repeated closed head impacts to assess the therapeutic efficacy of a monoclonal antibody (mAb) that blocks CGRP. To validate the model, we first assessed and optimized the development of periorbital and plantar tactile hypersensitivity in outbred CD1 mice. We then tested these responses after intraperitoneal injection of two migraine triggers: CGRP and sodium nitroprusside (SNP), a nitric oxide donor. Then, we assessed the efficacy of the anti-CGRP mAb using different administration paradigms.

**Results:**

Early administration of an anti-CGRP mAb before induction of TBI did not fully prevent the initial transient periorbital tactile hypersensitivity observed following multiple closed head injuries. However, the mAb did partially reduce persistent periorbital tactile hypersensitivity to a sub-threshold trigger. Administration of the mAb immediately after the injuries was also able to partially reduce persistent hypersensitivity. Importantly, injection of the anti-CGRP mAb 24 h prior to injection of a sub-threshold dose of CGRP or SNP fully prevented periorbital hypersensitivity to these triggers during the persistent sensitivity phase.

**Conclusions:**

These results indicate that an anti-CGRP mAb can partially, but not fully, attenuate cephalic tactile hypersensitivity when administered immediately before or after the mTBI event. In contrast, a stronger rescue was seen when the anti-CGRP mAb was administered just prior to CGRP and nitric oxide triggers. Thus, depending on the timing of administration, the anti-CGRP mAb can block persistent sensitization to headache triggers after mTBI.

**Supplementary Information:**

The online version contains supplementary material available at 10.1186/s10194-025-02108-x.

## Introduction

Mild traumatic brain injury (mTBI) is a global healthcare problem and one of the primary causes of disability worldwide [1]. The CDC reported over 214,000 TBI-related hospitalizations in 2020, and over 69,000 TBI-related deaths in 2021 [2]. Pain, including post-traumatic headache (PTH), is a common complication of TBI, which appears to be independent of psychologic disorders also prevalent in TBI patients [3]. The incidence of chronic pain and PTH in mTBI patients has been reported to be 51% and 75%, respectively [3]. Thus, new therapeutic approaches are desperately needed.

The management of TBI is currently based on the symptomatology experienced by the patient: anti-epileptic drugs for post-traumatic seizures, antidepressants for depression symptoms, antipsychotics for other neuropsychiatric disorders, etc [4]. To this day, no medications have been approved specifically for the treatment of PTH, and the pain and headache experienced by TBI patients remains poorly managed [5]. Because TBI-induced pain and PTH symptomatology resemble that of migraine [5], calcitonin gene-related peptide (CGRP) and its receptors are being studied as a potential target for the treatment of pain resulting from TBI events. In support of this avenue of research, eight CGRP-based therapeutics have been FDA-approved for acute and preventative treatment of migraine [6–8]. Indeed, a possible role for CGRP in PTH is supported by a recent study that showed patients become hypersensitive to infusions of CGRP after mTBI [9] and another study that reported low CGRP plasma levels in persistent PTH [10]. As a result, clinical trials investigating the efficacy of CGRP targeting drugs such as CGRP monoclonal antibodies (mAbs) are ongoing, but preliminary results are conflicting, with some reporting an improvement in patient symptoms after starting the treatment [11, 12], and others reporting no superior efficacy of the antibodies compared to placebo [13].

This difference in preliminary results is not surprising given the variability in clinical presentation seen in TBI patients, which results from the large heterogeneity of TBI events (e.g. localization of the primary impact, force of the impact, focal or diffuse modality, etc.). Patients with TBI and PTH will likely respond differently to treatments depending on the injury they received and the chronicity of their illness. Dissecting this heterogeneity in clinic will be difficult. However, animal models of TBI allow us to use a single type of injury to gain some insight into specific types of injuries and how they could respond to different treatments. In a previous study, we described a model of repeated mild closed head injury in which the TBI events resulted in an acute, transient tactile sensitivity in mice, followed by a persistent hypersensitization to non-noxious migraine triggers at very low doses [14]. In the present study, we used this model to assess the efficacy of a CGRP mAb to prevent those symptoms in male and female mice. This CGRP mAb (ALD405) has previously been shown to be efficacious for treating migraine-like symptoms of light aversion [15], tactile allodynia [16], spontaneous (unevoked) pain [17], and diarrhea [18] in mouse models.

## Methods

### Animals

Wildtype outbred CD1 mice were obtained from Charles River Laboratories, Roanoke, IL at 8 weeks of age and acclimated for one week in the animal facility prior to experimental testing. Equal numbers of male and female mice were used. Estrous cycle was not determined for any mice. Mice were housed 4 per cage on a 12 h light cycle with food and water ad libitum. All experiments were performed between 8 AM and 3 PM, with mice randomized and investigators blinded to injury status and/or drug treatment as described in the Experimental Design section. Animal procedures were approved by the University of Iowa Animal Care and Use Committee and performed in accordance with standards set by the National Institutes of Health, policies of the International Association for the Study of Pain and ARRIVE guidelines. We have used a total of 470 mice in this study.

### Drug administration

All injections were performed intraperitoneally (i.p.) with a 0.3 mm × 13 mm needle. Dulbecco PBS (Hyclone) was used as the diluent and vehicle. The amounts injected were as follows: 0.01, 0.05, or 0.1 mg/kg rat α-CGRP (Sigma-Aldrich, St Louis, MO), 0.25, 1, or 2.5 mg/kg sodium nitroprusside (SNP) (Sigma-Aldrich, St Louis, MO), 30 mg/kg anti-CGRP monoclonal antibody (ALD405) or 30 mg/kg monoclonal IgG control antibody (AD26-10). The antibodies were provided by Lundbeck. Animals were gently handled so that no anesthetic agents were needed during injections. All injections were performed by either WCC, AK, or ASW. For induction of injuries, mice were anesthetized using inhaled 5% isoflurane.

### Closed head impact TBI

A previously described model of weight drop-mediated TBI was used [14]. Mice were anesthetized with 5% isoflurane for ~ 2 min, then immediately placed on a foam cushion in a head trauma device. The instrument consists of a fiberglass tube (80 cm high, inner diameter 13 mm), placed vertically over and lightly touching the head. A 30 g metal weight was then dropped from the top of the tube to strike the head at the temporal right side between the corner of the eye and the ear. When dropped from the top of the 80 cm tube, the 30 g weight generates a force of 0.294 N when it hits the skull. The foam cushion prevented rotation of the head. Immediately after impact, mice were placed in their home cage and observed until their righting reflex returned. Animals received 3 TBI procedures, with one procedure per day over 3 days. There were no visible physical signs of trauma in any of the animals exposed to TBI. We previously reported that there were no detectable skull fractures or lesions following postmortem analysis [14].

### Periorbital and plantar tactile sensitivity

For periorbital sensitivity, mice were tested as described by Avona et al. [19], with slight modifications [14]. Each mouse was acclimated to its own polycoated paper cup (Choice 4 oz. paper cups; 6.5 cm top diameter, 4.5 cm bottom diameter, 72.5 cm length) for 20 min each day for 5 to 10 days, until habituated. Cups were placed on the edge of a platform about 8 inches above the table surface during habituation and testing. During each acclimation period, the D (0.07 g) von Frey filament was repeatedly approached to the periorbital area to just lightly touch the head without applying pressure, in order to decrease their withdrawal reflex. Mice were considered habituated once the tip of the filament could reach the head (without any pressure) without the mouse reacting to it. For plantar testing, mice were habituated to an acrylic chamber (dimensions 114 × 80 cm) placed over a grid support (Bioseb, France), for 2 h on the day before the first testing, and for 30 min immediately before testing. Habituations were done by different individuals than the experimenter. For habituations and testing, mice were tested a cage at a time so that both sexes were tested sequentially. The same cups and chambers were used throughout the experiment and the chambers were cleaned, but cups were not.

Periorbital and plantar von Frey tests were done on different days. Animals were tested sequentially at each respective time point following injection. For example, a cage of four mice would be injected allowing 1.5–2 min of testing per animal, so 6–8 min after injections, we would begin injecting mice in the next cage and stop injections once we had enough mice to test in a full 15 min window prior to testing in the next 15 min window.

A set of eight von Frey filaments was used from A (0.008 g) to H (1 g) (Bioseb, France). Testing was performed according to the up and down method previously described [20, 21]. Prior to testing, mice were given an hour to acclimate to the testing room. The injections were performed by an experienced technician with the ability to quickly scruff, inject, and re-cage the animal causing minimal distress to the animal. Filaments were applied for 3 s at the periorbital area above the eyes on the midline, or for 5 s at the plantar area. The D (0.07 g) filament was applied first. If the animal reacted (withdrew the head, wiped eyes or periorbital region, withdrew, shook, or licked hindpaw for plantar), then a lower filament was applied. If there was no reaction, then a higher filament was applied. A pattern was recorded. This method was used until 5 applications after a first change in the pattern were assessed. The last filament that was applied and the final pattern were used to calculate the 50% threshold following an established Eqs. [20, 21]. Since this technique does not yield continuous thresholds and data cannot be analyzed using parametric statistics, the 50% thresholds (g) were log-transformed before being analyzed in order to obtain normally distributed data.

### Experimental design

For each experiment, male and female mice were brought to the behavioral room for acclimation for 1 h prior to handling/testing. Males and females were tested in the same experiments. For the CGRP and SNP dosage experiments, baseline 50% thresholds were obtained 2–4 days prior to treatments. For the TBI experiments, baseline 50% thresholds were recorded for each animal 4 days before first TBI injury. Animals were then separated into experimental groups using a block randomization protocol per cage. All 4 animals within a same cage were allocated to the same injury group in case they develop anxiety from having an injured mouse in their group. All subsequent time-points are expressed as days after the last injury exposure. When treatments were administered, animals were placed back into their home cages immediately after drug administration and before the beginning of testing. Mice were tested 30 min after CGRP and 1 h after SNP administration. The mAb injections were done depending on the specific experiment, as described in the appropriate figure legends.

### Statistical analysis

Data were analyzed over both time and between individuals as in a previous study [14], using ANOVA tests as reported in Table 1 and Supplementary Table 1. When data were plotted as a function of time (line graphs) a two-way repeated measures ANOVA was performed (factors time and treatment), a Tukey multiple-comparison test was performed to compare all pairs of treatments at each time point when the overall test in the model was significant as detailed in Table 1. Note that the Tukey test only adjusted p-values for the treatment comparisons within each specific time-point. For peak effect time points data were presented in scatter plots to show the variability between mice. For individual mouse data (scatter plots), either a one-way ANOVA was performed (factor treatment), or a two-way ANOVA (factors treatment and antibody), followed by a Tukey multiple-comparison test to compare between treatments. Most tests met assumptions for ANOVA testing, with a few exceptions that had 1–2 groups lacking normality. Since these cases had mixed data sets, we elected to use the same test for all the data instead of using a mixture of ANOVA and nonparametric Kruskal-Wallis tests. For the scatter plot analyses with only two groups, because an ANOVA was not possible, t tests were used. Note that all statistical tests were unpaired. A power analysis was performed for sample size estimation using ClinCalc.com. The effect size of this study was estimated at 50% decrease for tactile hypersensitivity, based on data from previous similar studies. With an alpha of 0.05 and power at 0.80, the projected sample size needed with this effect size was approximately 14 per group for tactile hypersensitivity. This power analysis was confirmed in our previous experiments and publication [14]. Data are reported as mean ± standard error of the mean (SEM). Data were analyzed using GraphPad Prism 10.4.0 software (Graphpad.com). Significance was set at *p* < 0.05. The effect size was powered for analysis of males and females pooled together. Sex differences were observed in some experiments, so data separated by sex are also presented. In some experiments, sex separated analyses were not adequately powered, as indicated. Exclusions were applied to the datasets for the following reasons: fight wounds or development of dermatitis, which could have affected pain thresholds. All data from those mice (*n* = 7) were excluded from the study. In the experiments with mAb injections 1 day prior to testing sub-threshold triggers, eight mice from each treatment group on days 14 and 15 were excluded from analysis due to the disruptive effects of construction noise. The noise caused the mice to become hyperactive, making them untestable during these days. There was no animal loss from the closed head impact procedure.

**Table 1:** Statistical analyses

Figure #	Analysis	Statistics (symbol on Figure)
Figure 1A	Two-way repeated measure ANOVA	
	Interaction factor	*F*_(18,336)_=8.069, *p<*0.0001
	Treatment factor	*F*_(3,56)_=7.492, *p=*0.0003
	Time factor	*F*_(4.320,241.9)_=37.27, *p<*0.0001
	Tukey’s multiple comparison	
	at 15 min.	
	- Veh vs. CGRP 0.01mg/kg	*p=*0.8690
	- Veh vs. CGRP 0.05mg/kg	*p=*0.0012 (**)
	- Veh vs. CGRP 0.1mg/kg	*p=*0.0009 (***)
	- CGRP 0.01mg/kg vs. CGRP 0.05mg/kg	*p=*0.0207
	- CGRP 0.01mg/kg vs. CGRP 0.1mg/kg	*p=*0.0125
	- CGRP 0.05mg/kg vs. CGRP 0.1mg/kg	*p=*0.9865
	at 30 min.	
	- Veh vs. CGRP 0.01mg/kg	*p*=0.8270
	- Veh vs. CGRP 0.05mg/kg	*p*=0.0001 (***)
	- Veh vs. CGRP 0.1mg/kg	*p*<0.0001 (****)
	- CGRP 0.01mg/kg vs. CGRP 0.05mg/kg	*p*=0.0022
	- CGRP 0.01mg/kg vs. CGRP 0.1mg/kg	*p*=0.0017
	- CGRP 0.05mg/kg vs. CGRP 0.1mg/kg	*p*>0.9999
	at 45 min.	
	- Veh vs. CGRP 0.01mg/kg	*p*=0.8515
	- Veh vs. CGRP 0.05mg/kg	*p*=0.0001 (***)
	- Veh vs. CGRP 0.1mg/kg	*p*=0.0002 (***)
	- CGRP 0.01mg/kg vs. CGRP 0.05mg/kg	*p*=0.0024
	- CGRP 0.01mg/kg vs. CGRP 0.1mg/kg	*p*=0.0044
	- CGRP 0.05mg/kg vs. CGRP 0.1mg/kg	*p*=0.9946
	at 60 min.	
	- Veh vs. CGRP 0.01mg/kg	*p*=0.9691
	- Veh vs. CGRP 0.05mg/kg	*p*=0.2231
	- Veh vs. CGRP 0.1mg/kg	*p*=0.0208 (*)
	- CGRP 0.01mg/kg vs. CGRP 0.05mg/kg	*p*=0.6289
	- CGRP 0.01mg/kg vs. CGRP 0.1mg/kg	*p*=0.1141
	- CGRP 0.05mg/kg vs. CGRP 0.1mg/kg	*p*=0.6271
	at 75 min.	
	- Veh vs. CGRP 0.01mg/kg	*p*=0.8156
	- Veh vs. CGRP 0.05mg/kg	*p*=0.0857
	- Veh vs. CGRP 0.1mg/kg	*p*=0.0387 (*)
	- CGRP 0.01mg/kg vs. CGRP 0.05mg/kg	*p*=0.5747
	- CGRP 0.01mg/kg vs. CGRP 0.1mg/kg	*p*=0.3800
	- CGRP 0.05mg/kg vs. CGRP 0.1mg/kg	*p*=0.9847
	at 90 min.	
	- Veh vs. CGRP 0.01mg/kg	*p*=0.6998
	- Veh vs. CGRP 0.05mg/kg	*p*=0.2489
	- Veh vs. CGRP 0.1mg/kg	*p*=0.2267
	- CGRP 0.01mg/kg vs. CGRP 0.05mg/kg	*p*=0.9457
	- CGRP 0.01mg/kg vs. CGRP 0.1mg/kg	*p*=0.8062
	- CGRP 0.05mg/kg vs. CGRP 0.1mg/kg	*p*=0.9704
Figure 1B	One-way ANOVA	*F*_(3,56)_=14.36, *p<*0.0001
	Tukey’s multiple comparison	
	- Veh vs. CGRP 0.01mg/kg	*p*=0.8910
	- Veh vs. CGRP 0.05mg/kg	*p*<0.0001 (****)
	- Veh vs. CGRP 0.1mg/kg	*p*<0.0001 (****)
	- CGRP 0.01mg/kg vs. CGRP 0.05mg/kg	*p*=0.0006 (###)
	- CGRP 0.01mg/kg vs. CGRP 0.1mg/kg	*p*=0.0005 (###)
	- CGRP 0.05mg/kg vs. CGRP 0.1mg/kg	*p*>0.9999
Figure 1C	One-way ANOVA	*F*_(3,56)_=20.88, *p<*0.0001
	Tukey’s multiple comparison	
	- Veh vs. CGRP 0.01mg/kg	*p*=0.5535
	- Veh vs. CGRP 0.05mg/kg	*p*<0.0001 (****)
	- Veh vs. CGRP 0.1mg/kg	*p*<0.0001 (****)
	- CGRP 0.01mg/kg vs. CGRP 0.05mg/kg	*p*=0.0065 (##)
	- CGRP 0.01mg/kg vs. CGRP 0.1mg/kg	*p*=0.0002 (###)
	- CGRP 0.05mg/kg vs. CGRP 0.1mg/kg	*p*=0.9438
Figure 2A	Two-way repeated measure ANOVA	
	Interaction factor	*F*_(9,174)_=8.905, *p*<0.0001
	Treatment factor	*F*_(3,58)_=30.48, *p*<0.0001
	Time factor	*F*_(2.453,147.5)_=19.94, *p*<0.0001
	Tukey’s multiple comparison	
	at 30 min.	
	- Veh vs. SNP 0.25mg/kg	*p*=0.9973
	- Veh vs. SNP 1mg/kg	*p*=0.1958
	- Veh vs. SNP 2.5mg/kg	*p*<0.0001 (****)
	- SNP 0.25mg/kg vs. SNP 1mg/kg	*p*=0.2734
	- SNP 0.25mg/kg vs. SNP 2.5mg/kg	*p*<0.0001 (####)
	- SNP 1mg/kg vs. SNP 2.5mg/kg	*p*=0.0007 (^^^)
	at 60 min.	
	- Veh vs. SNP 0.25mg/kg	*p*=0.9889
	- Veh vs. SNP 1mg/kg	*p*=0.6343
	- Veh vs. SNP 2.5mg/kg	*p*<0.0001 (****)
	- SNP 0.25mg/kg vs. SNP 1mg/kg	*p*=0.6459
	- SNP 0.25mg/kg vs. SNP 2.5mg/kg	*p*<0.0001 (####)
	- SNP 1mg/kg vs. SNP 2.5mg/kg	*p*<0.0001 (^^^^)
	at 90 min.	
	- Veh vs. SNP 0.25mg/kg	*p*=0.7387
	- Veh vs. SNP 1mg/kg	*p*=0.7485
	- Veh vs. SNP 2.5mg/kg	*p*<0.0001 (****)
	- SNP 0.25mg/kg vs. SNP 1mg/kg	*p*>0.9999
	- SNP 0.25mg/kg vs. SNP 2.5mg/kg	*p*=0.0014 (##)
	- SNP 1mg/kg vs. SNP 2.5mg/kg	*p*=0.0011 (^^)
Figure 2B	One-way ANOVA	*F*_(3,58)_=32.53, *p*<0.0001
	Tukey’s multiple comparison	
	- Veh vs. SNP 0.25mg/kg	*p*=0.9929
	- Veh vs. SNP 1mg/kg	*p*=0.7171
	- Veh vs. SNP 2.5mg/kg	*p*<0.0001 (****)
	- SNP 0.25mg/kg vs. SNP 1mg/kg	*p*=0.8533
	- SNP 0.25mg/kg vs. SNP 2.5mg/kg	*p*<0.0001 (####)
	- SNP 1mg/kg vs. SNP 2.5mg/kg	*p*<0.0001 (^^^^)
Figure 2C	One-way ANOVA	*F*_(3,58)_=7.279, *p*=0.0003
	Tukey’s multiple comparison	
	- Veh vs. SNP 0.25mg/kg	*p*=0.8290
	- Veh vs. SNP 1mg/kg	*p*=0.7160
	- Veh vs. SNP 2.5mg/kg	*p*=0.0193 (*)
	- SNP 0.25mg/kg vs. SNP 1mg/kg	*p*=0.9961
	- SNP 0.25mg/kg vs. SNP 2.5mg/kg	*p*=0.0013 (##)
	- SNP 1mg/kg vs. SNP 2.5mg/kg	*p*=0.0008 (^^^)
Figure 3A	Two-way repeated measure ANOVA	
	Interaction factor	*F*_(9,201)_=0.6281, *p*=0.7723
	Treatment factor	*F*_(3,67)_=35.96, *p*<0.0001
	Time factor	*F*_(2.491,166.9)_=0.5489, *p*=0.6170
	Tukey’s multiple comparison at day 28	
	- Con mAb+PBS vs. Con mAb+CGRP	*p*<0.0001 (****)
	- Con mAb+PBS vs. CGRP mAb+PBS	*p*=0.5524
	- Con mAb+PBS vs. CGRP mAb+CGRP	*p*=0.0160
	- Con mAb+CGRP vs. CGRP mAb+PBS	*p*<0.0001
	- Con mAb+CGRP vs. CGRP mAb+CGRP	*p*=0.0643
	- CGRP mAb+PBS vs. CGRP mAb+CGRP	*p*=0.0012 (**)
	Tukey’s multiple comparison at day 45	
	- Con mAb+PBS vs. Con mAb+CGRP	*p*<0.0001 (****)
	- Con mAb+PBS vs. CGRP mAb+PBS	*p*=0.7492
	- Con mAb+PBS vs. CGRP mAb+CGRP	*p*=0.0013
	- Con mAb+CGRP vs. CGRP mAb+PBS	*p*<0.0001
	- Con mAb+CGRP vs. CGRP mAb+CGRP	*p*=0.4430
	- CGRP mAb+PBS vs. CGRP mAb+CGRP	*p*=0.0066 (**)
	Tukey’s multiple comparison at day 65	
	- Con mAb+PBS vs. Con mAb+CGRP	*p*<0.0001 (****)
	- Con mAb+PBS vs. CGRP mAb+PBS	*p*=0.7130
	- Con mAb+PBS vs. CGRP mAb+CGRP	*p*=0.0006
	- Con mAb+CGRP vs. CGRP mAb+PBS	*p*=0.0004
	- Con mAb+CGRP vs. CGRP mAb+CGRP	*p*=0.1162
	- CGRP mAb+PBS vs. CGRP mAb+CGRP	*p*=0.0184 (*)
	Tukey’s multiple comparison at day 80	
	- Con mAb+PBS vs. Con mAb+CGRP	*p*<0.0001 (****)
	- Con mAb+PBS vs. CGRP mAb+PBS	*p*=0.9971
	- Con mAb+PBS vs. CGRP mAb+CGRP	*p*=0.0035
	- Con mAb+CGRP vs. CGRP mAb+PBS	*p*=0.0001
	- Con mAb+CGRP vs. CGRP mAb+CGRP	*p*=0.0081 (^^)
	- CGRP mAb+PBS vs. CGRP mAb+CGRP	*p*=0.1088
Figure 3B Left panel	t test	*p*=0.0113 (*)
Figure 3B Right panel	t test	*p*=0.4068
Figure 3C Left panel	Two-way ANOVA	
	Interaction factor	*F*_(1,67)_=2.759, *p*=0.1014
	Treatment factor	*F*_(1,67)_=53.12, *p*<0.0001
	Antibody factor	*F*_(1,67)_=8.551, *p*=0.0047
	Tukey’s multiple comparison	
	- Con mAb+PBS vs. Con mAb+CGRP	*p*<0.0001 (****)
	- Con mAb+PBS vs. CGRP mAb+PBS	*p*=0.8050
	- Con mAb+PBS vs. CGRP mAb+CGRP	*p*=0.0162
	- Con mAb+CGRP vs. CGRP mAb+PBS	*p*<0.0001
	- Con mAb+CGRP vs. CGRP mAb+CGRP	*p*=0.0104 (^)
	- CGRP mAb+PBS vs. CGRP mAb+CGRP	*p*=0.0011 (**)
Figure 3C Right panel	Two-way ANOVA	
	Interaction factor	*F*_(1,67)_=6.415, *p*=0.0137
	Treatment factor	*F*_(1,67)_=37.59, *p*<0.0001
	Antibody factor	*F*_(1,67)_=5.053, *p*=0.0279
	Tukey’s multiple comparison	
	- Con mAb+PBS vs. Con mAb+CGRP	*p*<0.0001 (****)
	- Con mAb+PBS vs. CGRP mAb+PBS	*p*=0.9970
	- Con mAb+PBS vs. CGRP mAb+CGRP	*p*=0.0398
	- Con mAb+CGRP vs. CGRP mAb+PBS	*p*<0.0001
	- Con mAb+CGRP vs. CGRP mAb+CGRP	*p*=0.0070 (^^)
	- CGRP mAb+PBS vs. CGRP mAb+CGRP	*p*=0.0650
Figure 4A	Two-way mixed-effects ANOVA	
	Interaction factor	*F*_(6,111)_=2.429, *p*=0.0304
	Treatment factor	*F*_(3,58)_=28.14, *p*<0.0001
	Time factor	*F*_(1.984,110.1)_=4.265, *p*=0.0167
	Tukey’s multiple comparison at day 28	
	- Con mAb+PBS vs. Con mAb+CGRP	*p*<0.0001 (****)
	- Con mAb+PBS vs. CGRP mAb+PBS	*p*=0.9995
	- Con mAb+PBS vs. CGRP mAb+CGRP	*p*=0.0080
	- Con mAb+CGRP vs. CGRP mAb+PBS	*p*<0.0001
	- Con mAb+CGRP vs. CGRP mAb+CGRP	*p*=0.1062
	- CGRP mAb+PBS vs. CGRP mAb+CGRP	*p*=0.0081 (**)
	Tukey’s multiple comparison at day 60	
	- Con mAb+PBS vs. Con mAb+CGRP	*p*=0.0390 (*)
	- Con mAb+PBS vs. CGRP mAb+PBS	*p*=0.6645
	- Con mAb+PBS vs. CGRP mAb+CGRP	*p*=0.0063
	- Con mAb+CGRP vs. CGRP mAb+PBS	*p*=0.0078
	- Con mAb+CGRP vs. CGRP mAb+CGRP	*p*=0.9938
	- CGRP mAb+PBS vs. CGRP mAb+CGRP	*p*=0.0004 (***)
	Tukey’s multiple comparison at day 80	
	- Con mAb+PBS vs. Con mAb+CGRP	*p*=0.2698
	- Con mAb+PBS vs. CGRP mAb+PBS	*p*=0.3127
	- Con mAb+PBS vs. CGRP mAb+CGRP	*p*=0.0007
	- Con mAb+CGRP vs. CGRP mAb+PBS	*p*=0.0323
	- Con mAb+CGRP vs. CGRP mAb+CGRP	*p*=0.5730
	- CGRP mAb+PBS vs. CGRP mAb+CGRP	*p*<0.0001 (****)
Figure 4B Left panel	t test	*p*=0.8802
Figure 4B Right panel	t test	*p*=0.0145 (*)
Figure 4C Left panel	Two-way ANOVA	
	Interaction factor	*F*_(1,56)_=3.943, *p*=0.0520
	Treatment factor	*F*_(1,56)_=49.42, *p*<0.0001
	Antibody factor	*F*_(1,56)_=3.510, *p*=0.0662
	Tukey’s multiple comparison	
	- Con mAb+PBS vs. Con mAb+CGRP	*p*<0.0001 (****)
	- Con mAb+PBS vs. CGRP mAb+PBS	*p*=0.9998
	- Con mAb+PBS vs. CGRP mAb+CGRP	*p*=0.0032
	- Con mAb+CGRP vs. CGRP mAb+PBS	*p*<0.0001
	- Con mAb+CGRP vs. CGRP mAb+CGRP	*p*=0.0322 (^)
	- CGRP mAb+PBS vs. CGRP mAb+CGRP	*p*=0.0041 (**)
Figure 4C Right panel	Two-way ANOVA	
	Interaction factor	*F*_(1,56)_=3.839, *p*=0.0551
	Treatment factor	*F*_(1,56)_=24.10, *p*<0.0001
	Antibody factor	*F*_(1,56)_=0.04188, *p*=0.8386
	Tukey’s multiple comparison	
	- Con mAb+PBS vs. Con mAb+CGRP	*P*=0.1711
	- Con mAb+PBS vs. CGRP mAb+PBS	*p*=0.6166
	- Con mAb+PBS vs. CGRP mAb+CGRP	*p*=0.0042
	- Con mAb+CGRP vs. CGRP mAb+PBS	*p*=0.0070
	- Con mAb+CGRP vs. CGRP mAb+CGRP	*p*=0.4115
	- CGRP mAb+PBS vs. CGRP mAb+CGRP	*p*<0.0001 (****)
Figure 5A Left panel	Two-way mixed-effects ANOVA	
	Interaction factor	*F*_(9,229)_=4.461, *p*<0.0001
	Treatment factor	*F*_(3,87)_=4.144, *p*=0.0085
	Time factor	*F*_(2.357,179.9)_=62.56, *p*<0.0001
	Tukey’s multiple comparison at day 14	
	- Con mAb+PBS vs. Con mAb+CGRP	*p*=0.0008 (***)
	- Con mAb+PBS vs. CGRP mAb+PBS	*p*>0.9999
	- Con mAb+PBS vs. CGRP mAb+CGRP	*p*=0.8538
	- Con mAb+CGRP vs. CGRP mAb+PBS	*p*=0.0006
	- Con mAb+CGRP vs. CGRP mAb+CGRP	*p*=0.0146 (^)
	- CGRP mAb+PBS vs. CGRP mAb+CGRP	*p*=0.8584
	Tukey’s multiple comparison at day 21	
	- Con mAb+PBS vs. Con mAb+CGRP	*p*<0.0001 (****)
	- Con mAb+PBS vs. CGRP mAb+PBS	*p*=0.9597
	- Con mAb+PBS vs. CGRP mAb+CGRP	*p*=0.4116
	- Con mAb+CGRP vs. CGRP mAb+PBS	*p*<0.0001
	- Con mAb+CGRP vs. CGRP mAb+CGRP	*p*=0.0952
	- CGRP mAb+PBS vs. CGRP mAb+CGRP	*p*=0.2312
Figure 5A Right panel	Two-way repeated measure ANOVA	
	Interaction factor	*F*_(1,55)_=7.262, *p*=0.0093
	Treatment factor	*F*_(1,55)_=14.30, *p*=0.0004
	Antibody factor	*F*_(1,55)_=7.047, *p*=0.0104
	Tukey’s multiple comparison	
	- PBS:Con mAb vs. PBS:CGRP mAb	*p*>0.9999
	- PBS:Con mAb vs. CGRP:Con mAb	*p*=0.0003 (***)
	- PBS:Con mAb vs. CGRP: CGRP mAb	*p*=0.8952
	- PBS:CGRP mAb vs. CGRP:Con mAb	*p*=0.0001
	- PBS:CGRP mAb vs. CGRP: CGRP mAb	*p*=0.8525
	- CGRP:Con mAb vs. CGRP: CGRP mAb	*p*=0.0019 (^^)
Figure 5B Left panel	Two-way mixed-effects ANOVA	
	Interaction factor	*F*_(9,229)_=4.857, *p<*0.0001
	Treatment factor	*F*_(3,87)_=3.987, *p*=0.0104
	Time factor	*F*_(2.514,191.9)_=59.48, *p<*0.0001
	Tukey’s multiple comparison at day 15	
	- Con mAb+PBS vs. Con mAb+SNP	*p=*0.0069 (**)
	- Con mAb+PBS vs. CGRP mAb+PBS	*p>*0.9999
	- Con mAb+PBS vs. CGRP mAb+SNP	*p=*0.5248
	- Con mAb+SNP vs. CGRP mAb+PBS	*p=*0.0065
	- Con mAb+SNP vs. CGRP mAb+SNP	*p=*0.1255
	- CGRP mAb+PBS vs. CGRP mAb+SNP	*p=*0.5277
	Tukey’s multiple comparison at day 22	
	- Con mAb+PBS vs. Con mAb+SNP	*p*<0.0001 (****)
	- Con mAb+PBS vs. CGRP mAb+PBS	*p*=0.8654
	- Con mAb+PBS vs. CGRP mAb+SNP	*p*=0.1215
	- Con mAb+SNP vs. CGRP mAb+PBS	*p*<0.0001
	- Con mAb+SNP vs. CGRP mAb+SNP	*p*=0.1333
	- CGRP mAb+PBS vs. CGRP mAb+SNP	*p*=0.2948
Figure 5B Right panel	Two-way repeated measure ANOVA	
	Interaction factor	*F*_(1,55)_=3.782, *p=*0.0569
	Treatment factor	*F*_(1,55)_=13.69, *p*=0.0005
	Antibody factor	*F*_(1,55)_=3.570, *p=*0.0641
	Tukey’s multiple comparison	
	- PBS:Con mAb vs. PBS:CGRP mAb	*p>*0.9999
	- PBS:Con mAb vs. SNP:Con mAb	*p=*0.0018 (**)
	- PBS:Con mAb vs. SNP: CGRP mAb	*p=*0.5870
	- PBS:CGRP mAb vs. SNP:Con mAb	*p*=0.0011
	- PBS:CGRP mAb vs. SNP: CGRP mAb	*p*=0.5680
	- SNP:Con mAb vs. SNP: CGRP mAb	*p*=0.0402 (^)

## Results

### Peripheral CGRP and SNP administrations induce periorbital tactile hypersensitivity in a dose-dependent manner in naive mice

**Fig. 1:**
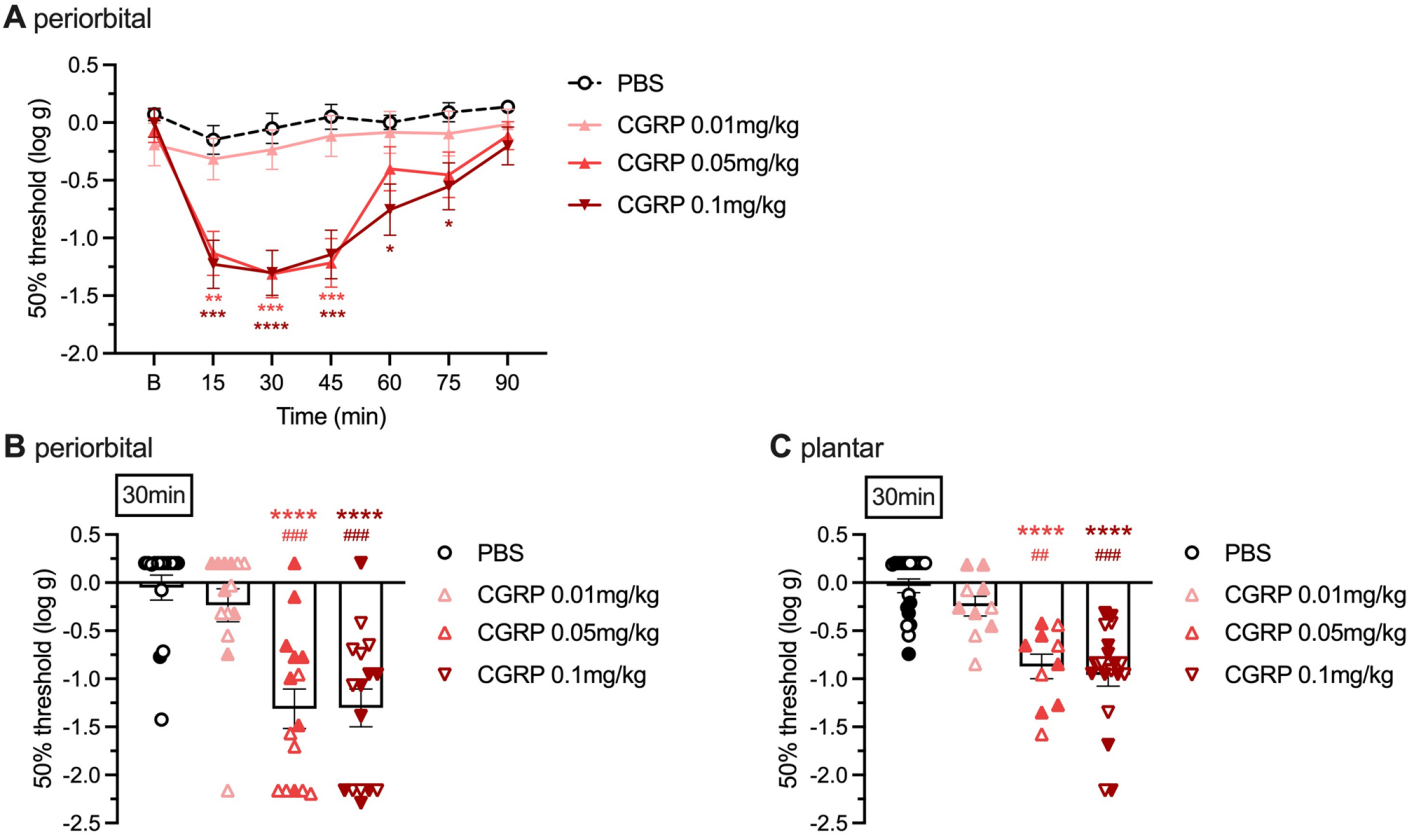
Development of periorbital and plantar tactile hypersensitivity after peripheral CGRP in CD1 mice. **A** Dose curve and time course of periorbital tactile hypersensitivity after i.p. CGRP injection. The threshold of von Frey filament sensitivity was assessed before injection at baseline (B), 2–4 days prior to treatments, and at the indicated time points after injection of PBS (*n* = 15), CGRP 0.01 mg/kg (*n* = 14), CGRP 0.05 mg/kg (*n* = 15), or CGRP 0.1 mg/kg (*n* = 16). **B** Scatter plot representation of the individual mice at 30 min. **C** Scatter plot representation of the hindpaw plantar hypersensitivity dose curve at 30 min after injection of PBS (*n* = 20), CGRP 0.01 mg/kg (*n* = 10), CGRP 0.05 mg/kg (*n* = 10), or CGRP 0.1 mg/kg (*n* = 20). The mean ± SEM 50% thresholds are shown. In scatter plot graph males are represented by open and females by closed symbols. For all panels *p < 0.05, **p < 0.01, ***p < 0.001, ****p < 0.0001 indicates significance compared with PBS, ##*p* < 0.01, ###*p* < 0.001 indicates significance compared with CGRP 0.01 mg/kg. Statistics are described in Table 1

Our previous publication reported that after repeated mTBI, mice first developed a transient periorbital tactile hypersensitivity followed by a persistent sensitization to normally non-noxious triggers such as sub-threshold doses of CGRP and SNP [14]. However, a longitudinal dose-response of the effect of peripheral CGRP and SNP administration on hypersensitivity in naive animals was never thoroughly performed. We therefore evaluated tactile sensitivity in naive CD1 mice, before and at different time-points after intraperitoneal administration of CGRP at 0.01, 0.05, and 0.1 mg/kg. Figure 1 shows a robust and significant decrease in the periorbital 50% thresholds in mice receiving 0.05 and 0.1 mg/kg of CGRP as early as 15 min after administration, with a peak effect at 30 min, and lasting between 60 and 75 min before thresholds returned to baseline values (Fig. 1A). The dose of 0.01 mg/kg did not change the thresholds compared to mice administered with saline. Figure 1B details individual mouse results at the 30 min time point, and highlights male (empty symbols) and female (full symbols) responses. The statistical power was reduced for analyses by sex so we did not analyze sex independently.

We also assessed the plantar tactile response in naive CD1 mice after administration of the same doses of CGRP and found a significant decrease in the plantar 50% thresholds 30 min after administration at the doses of 0.05 and 0.1 mg/kg (Fig. 1C), although the amplitude of this response appeared less robust than the one seen at the periorbital region. The dose of 0.01 mg/kg failed to change the thresholds compared to the saline administered group. The plantar responses by sex are also presented in Fig. 1C with open symbols for males and closed symbols for females.

**Fig. 2:**
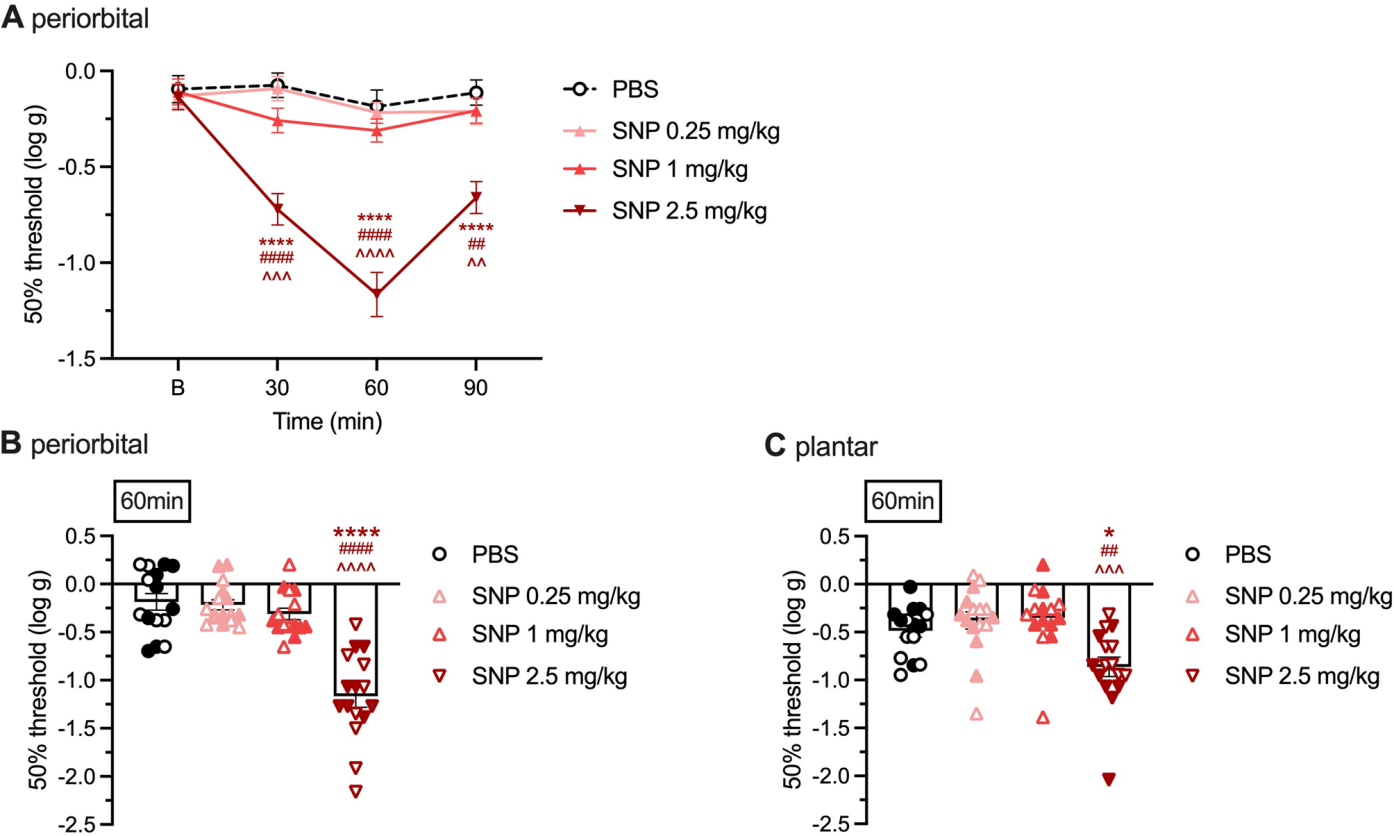
Development of periorbital and plantar tactile hypersensitivity after peripheral SNP in CD1 mice. **A** Dose curve and time course of periorbital tactile hypersensitivity after i.p. SNP injection. The threshold of von Frey filament sensitivity was assessed before injection at baseline (B), 2–4 days prior to treatments, and at the indicated times after injection of PBS (*n* = 15), SNP 0.25 mg/kg (*n* = 16), SNP 1 mg/kg (*n* = 15), or SNP 2.5 mg/kg (*n* = 16). **B** Scatter plot representation of the individual mice at 60 min. **C** Scatter plot representation of the hindpaw plantar hypersensitivity dose curve at 60 min after injection of PBS (*n* = 15), SNP 0.25 mg/kg (*n* = 16), SNP 1 mg/kg (*n* = 15), or SNP 2.5 mg/kg (*n* = 16). The mean ± SEM 50% thresholds are shown. In scatter plot graph males are represented by open and females by closed symbols. For all panels *p < 0.05, ****p < 0.0001 indicates significance compared with PBS, ##*p* < 0.01, ####*p* < 0.0001 indicates significance compared with SNP 0.25 mg/kg, ^^*p* < 0.01, ^^^*p* < 0.001, ^^^^*p* < 0.0001 indicates significance compared with SNP 1 mg/kg. Statistics are described in Table 1

We repeated the same experiments with SNP at the doses of 0.25, 1, and 2.5 mg/kg i.p. (Fig. 2). Peripheral administration of SNP induced a robust and significant decrease in 50% thresholds at the highest dose of 2.5 mg/kg, with peak effect at 60 min, while the two other doses did not change the thresholds (Fig. 2A). Figures 2B and C detail the individual mice responses 60 min after SNP administration, when tactile sensitivity is assessed in the periorbital and in the plantar area, respectively. Like CGRP-induced hypersensitivity, SNP-induced hypersensitivity appeared more pronounced in the periorbital area than in the plantar area. Data separated by sex is also presented with open symbols for males and closed symbols for females.

### Early administration of anti-CGRP mAb before closed head impacts does not rescue acute hypersensitivity but does partially reduce persistent periorbital tactile hypersensitivity

We used a model of mTBI-induced persistent hypersensitivity caused by 3 repeated closed head impact injuries over 3 consecutive days [14]. In this model, there is a transient, acute response of tactile sensitivity in the absence of any exogenous trigger followed by a prolonged, persistent hypersensitivity to sub-threshold triggers. Because periorbital hypersensitivity is more specific to headache and displayed a greater amplitude than plantar, the following experiments focused on periorbital testing.

The strategy was to test administration of the mAb at different times based on the rationale that an early treatment even before injury might be more effective than later treatments. For all experiments, we used only a single injection of the mAb to avoid potential confounders should there be an immune response to the humanized antibodies. In addition, a pilot test with one cohort of sham treated mice (not exposed to TBI) showed similar behavior with either the control or anti-CGRP mAb (not shown) as previously reported for sham mice [14]. Therefore, a sham group was not included in these studies in order to minimize the number of animals.

**Fig. 3:**
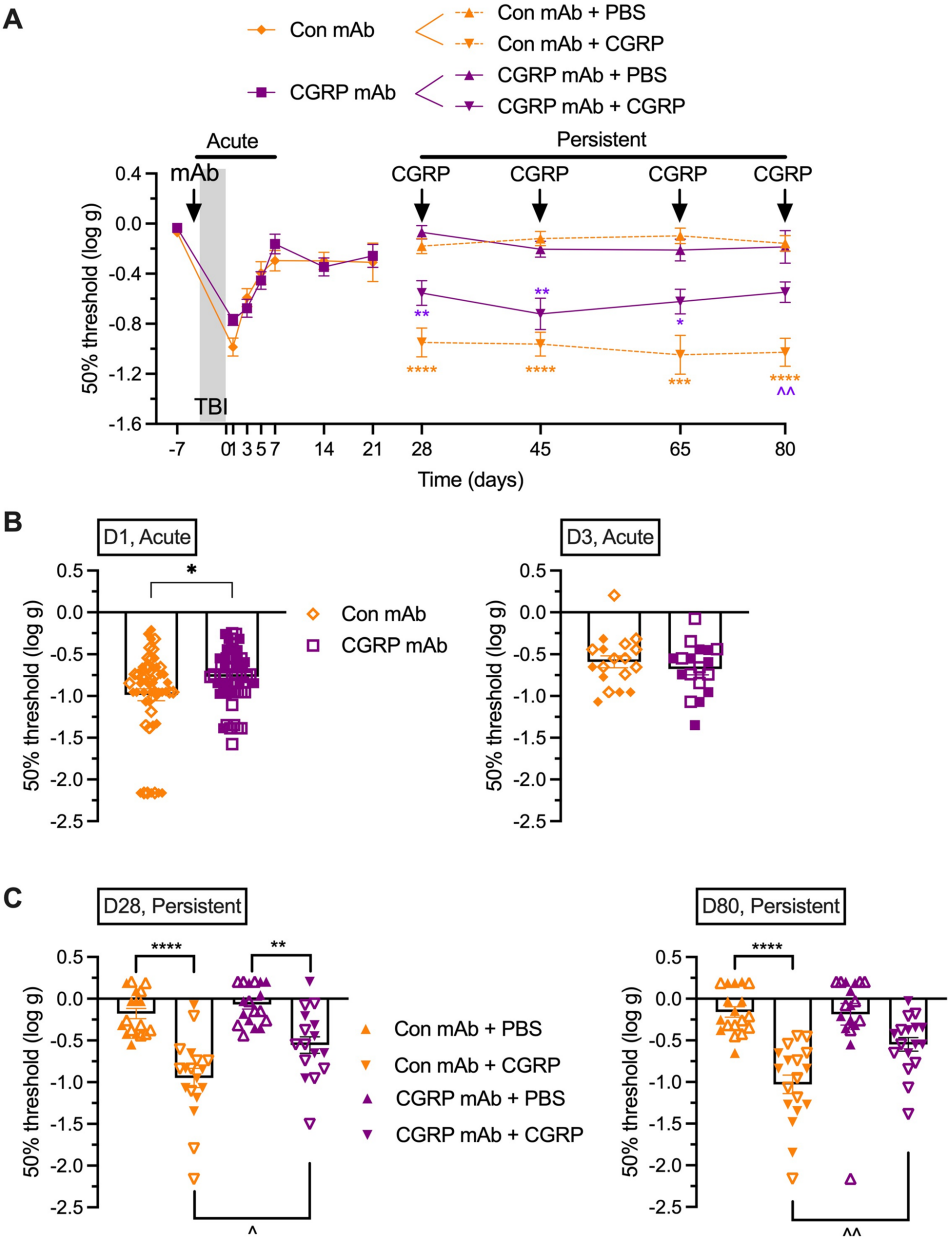
Injection of anti-CGRP mAb before mTBI does not prevent acute tactile sensitivity but partially rescues persistent hypersensitization. **A** Anti-CGRP mAb (CGRP mAb) or an isotype (Con) mAb was administered 24 h before first closed head impact injury. For the acute phase, periorbital tactile sensitivity was assessed at baseline (−7 days), 4 days before first injury, and at the indicated days after the last injury. TBI + Con mAb (*n* = 54 D-7 and D1, *n* = 18 D3-D21), TBI + CGRP mAb (*n* = 54 D-7 and D1, *n* = 18 D3-D21). For the persistent phase, the two cohorts were each split into two subgroups that received an i.p. injection of vehicle (PBS) or a sub-threshold dose of CGRP (0.01 mg/kg) on days 28, 45, 65 and 80 post-injury. TBI + Con mAb + PBS (*n* = 18), TBI + Con mAb + CGRP (*n* = 18), TBI + CGRP mAb + PBS (*n* = 18), TBI + CGRP mAb + CGRP (*n* = 17). The threshold of von Frey filament sensitivity was assessed 30 min after injection. **B** Scatter plot representation of the individual mice at day 1 and day 3. **C** Scatter plot representation of the individual mice at day 28 and day 80. The mean ± SEM 50% thresholds are presented in all graphs. In scatter plot graphs, males are represented by open and females by closed symbols. For panels A and C *p < 0.05, **p < 0.01, ***p < 0.001, ****p < 0.0001 indicates significance comparing CGRP and vehicle groups treated with same mAb, ^*p* < 0.05, ^^*p* < 0.01 indicates significance comparing two CGRP groups (treated with different mAb). For panel B *p < 0.05. Statistics are described in Table 1

Acute and persistent hypersensitivities were assessed by injecting an anti-CGRP mAb or an isotype control mAb 24 h prior to the first of 3 injuries (Fig. 3). Figure 3A shows the time-course of the effect of the anti-CGRP mAb in the acute (days 0 to 7) and persistent (days 28 to 80) periods compared to a control isotype mAb.

When administered before the injuries, the anti-CGRP mAb did not prevent acute sensitivity, as shown by the lack of differences between the anti-CGRP mAb group and the control mAb group (Fig. 3A, left side). Both groups had a robust decrease in the 50% thresholds compared to baseline levels obtained before the injury. It should be noted that when the individual mouse data were analyzed post-injury, there was a very small, but *p* < 0.05 difference between the control and anti-CGRP mAb groups at day 1, but not day 3 (Fig. 3B); however, the groups at baseline and day 1 had a relatively large number of mice compared to later time points since the groups were subdivided into different treatments after day 1 and the experiment was powered for those later time points. Thus, this difference at day 1, while *p* < 0.05, is unlikely to be biologically relevant. There were no differences between male and female mice, which are indicated as open symbols for males and full symbols for females.

To measure trigger-induced persistent hypersensitivity, the animals from each antibody group were divided into two groups at 28 days post-injury (Fig. 3A, right side). One group received PBS as a negative control, and the other group received a sub-threshold dose of CGRP. Among the mice given the isotype antibody before mTBI, the ones receiving sub-threshold CGRP showed a robust decrease in 50% thresholds, while the mice receiving PBS did not display any hypersensitivity. Mice that had been administered the anti-CGRP mAb showed a similar pattern, but the effect of sub-threshold CGRP was not as robust as in the isotype group. The difference from the isotype group is statistically significant at day 80 when data are viewed longitudinally, and at days 28 and 80 when the data are viewed as individual responses (Fig. 3C). This suggests a partial, but significant efficacy of the treatment to relieve persistent hypersensitivity lasting for at least 80 days after injuries. Of note, while the experiment did not have sufficient power to make statistical comparisons by sex, the data shown in Supplementary Fig. 1 indicate that this effect might be more pronounced in (if not driven by) females. This finding needs to be confirmed by experiments powered to show sex differences.

### Administration of anti-CGRP mAb immediately after closed head impacts partially reduces acute and persistent periorbital tactile hypersensitivity

**Fig. 4:**
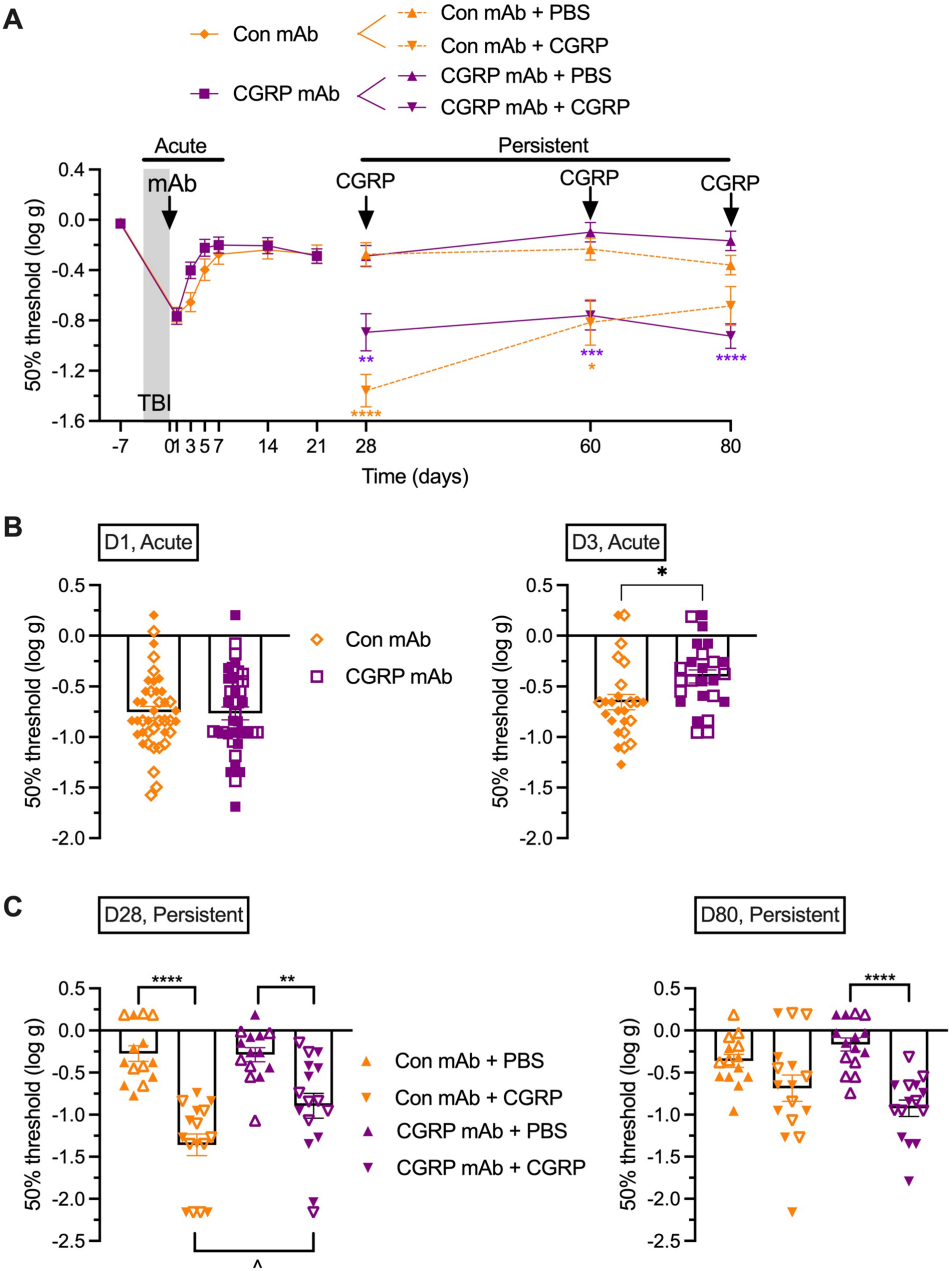
Injection of anti-CGRP mAb immediately after TBI partially reduces acute and persistent tactile hypersensitivity.** A** Anti-CGRP mAb (CGRP mAb) or an isotype (Con) mAb was administered right after the last closed head impact injury. For the acute phase, periorbital tactile sensitivity was assessed at baseline (−7 days), 4 days before first injury, and at the indicated days after the last injury. TBI + Con mAb (*n* = 41 D-7 and D1, *n* = 25 D3-D21), TBI + CGRP mAb (*n* = 41 D-7 and D1, *n* = 25 D3-D21). For the persistent phase, the two cohorts were each split into two subgroups that received an i.p. injection of vehicle (PBS) or a sub-threshold dose of CGRP (0.01 mg/kg) on days 28, 60 and 80 post-injury. TBI + Con mAb + PBS (*n* = 14), TBI + Con mAb + CGRP (*n* = 16), TBI + CGRP mAb + PBS (*n* = 15), TBI + CGRP mAb + CGRP (*n* = 16). The threshold of von Frey filament sensitivity was assessed 30 min after injection. **B** Scatter plot representation of the individual mice at day 1 and day 3. **C** Scatter plot representation of the individual mice at day 28 and day 80. The mean ± SEM 50% thresholds are presented in all graphs. In scatter plot graph males are represented by open and females by closed symbols. For panels A and C *p < 0.05, **p < 0.01, ***p < 0.001, ****p < 0.0001 indicates significance comparing CGRP and vehicle groups treated with same mAb. ^*p* < 0.05 indicates significance comparing two CGRP groups (treated with different mAb). For panel B *p < 0.05. Statistics are described in Table 1

The anti-CGRP mAb was then tested when administered immediately after the last impact injury, looking at acute (days 0 to 7) and persistent (days 28 to 80) hypersensitivities compared to a control isotype mAb (Fig. 4A). As with administration before injuries, the anti-CGRP mAb did not prevent acute sensitivity (Fig. 4A). However, when the data were analyzed as a scatter plot of individual responses, there was a *p* < 0.05 difference between the control and anti-CGRP mAb groups at day 3, but not day 1 (Fig. 4B). This suggests the anti-CGRP mAb treatment might be inducing a slightly faster recovery of the transient hypersensitivity after injuries. The lack of any rescue at day 1 may be due to delayed onset of the mAb, as compared to the previous experiment where mAb was given before mTBI and a small effect was seen on day 1. This effect seemed more pronounced in females than males (Supplementary Fig. 2), although this finding needs to be confirmed by experiments powered to show sex differences.

To measure trigger-induced persistent hypersensitivity, the animals from each antibody group were divided into two groups at 28 days post-injury (Fig. 4A, right side). One group received PBS as a negative control, and the other group received a sub-threshold dose of CGRP. Figure 4A shows that among the mice that had been administered with the isotype antibody after the TBI event, the ones receiving sub-threshold CGRP showed a robust decrease in 50% thresholds, while the mice receiving PBS did not display any hypersensitivity. Mice that had been administered the anti-CGRP mAb showed a similar pattern. However, at day 28 after injuries there was a partial rescue of the treatment, which was significant (Fig. 4C). The lack of significance at day 80 can be explained by either a lack of efficacy of the antibody at that time point, or, more likely, by the upward trend of the threshold for the control group that received the control antibody with CGRP, since the antibody-treated group showed a consistent 50% threshold level over time, similar to the previous experiment. Unfortunately, repeated von Frey testing in mice can sometimes desensitize the animals, which could explain this return to baseline. There was no difference between sexes at days 28 or 80.

### Administration of anti-CGRP mAb during the persistent phase of mTBI prevents periorbital hypersensitivity to migraine triggers

**Fig. 5:**
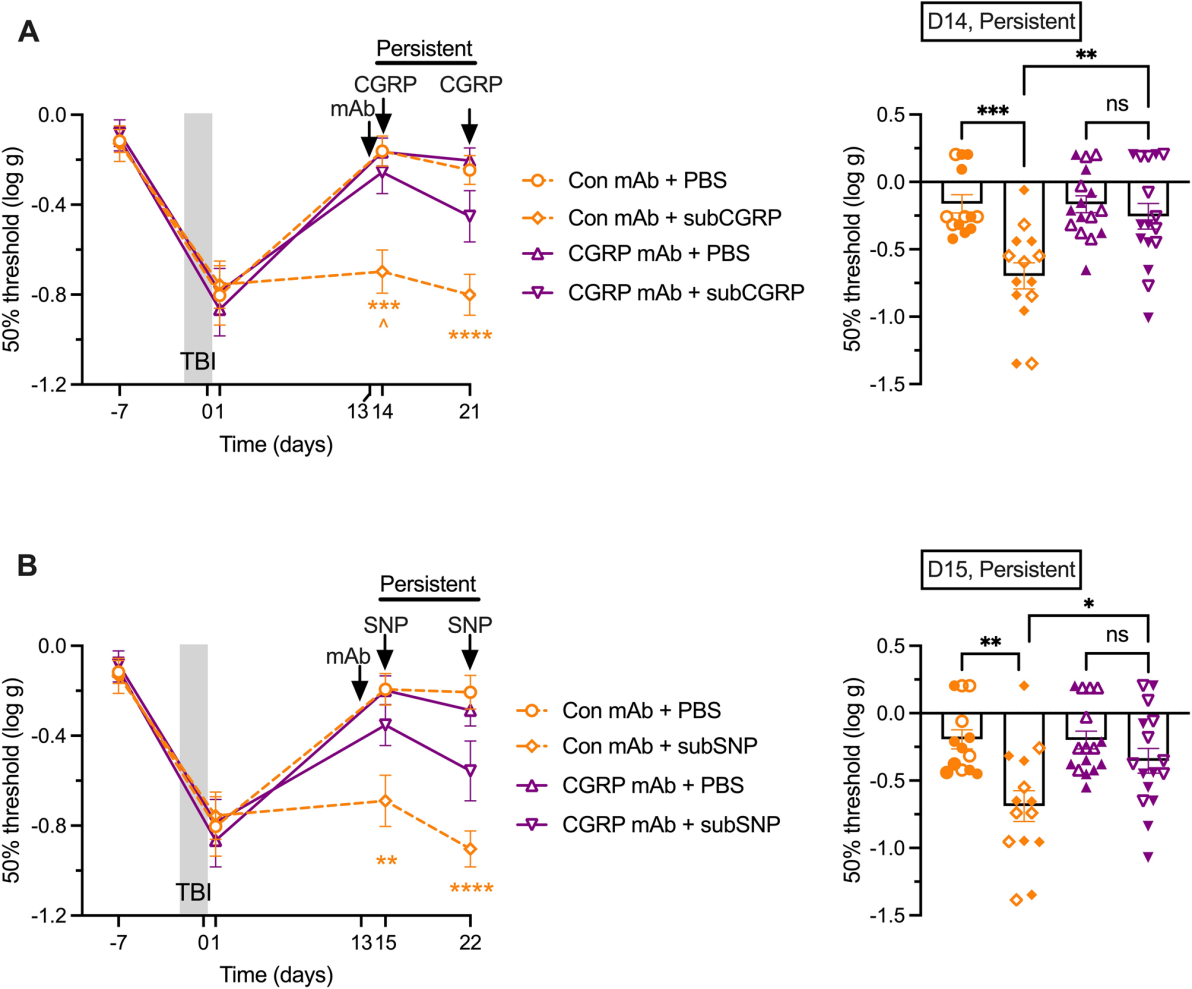
Injection of anti-CGRP mAb prior to a trigger prevents persistent tactile hypersensitivity after TBI. **A** Periorbital tactile acute sensitivity was assessed at baseline before injury (−7 days), 4 days before first injury, and at 1 day after the last closed head impact injury. At day 13, anti-CGRP mAb (CGRP mAb) or an isotype (Con) mAb was administered. The mice were then tested for persistent hypersensitivity on days 14 and 21 at 30 min after injection of vehicle (PBS) or sub-threshold dose of CGRP (0.01 mg/kg). Con mAb + PBS (*n* = 21 D-7, 1, 21, *n* = 13 D14), Con mAb + subCGRP (*n* = 22 D-7, 1, 21, *n* = 14 D14), CGRP mAb + PBS (*n* = 24 D-7, 1, 21, *n* = 16 D14), CGRP mAb + subCGRP (*n* = 24 D-7, 1, 21, *n* = 16 D14). Scatter plot representation of the individual mice at day 14. **B** Periorbital tactile hypersensitivity to SNP was assessed on days 15 and 22 on the same mice as in panel A, except after injection of vehicle (PBS) or sub-threshold dose of SNP (0.25 mg/kg). Con mAb + PBS (*n* = 21 D-7, 1, 21, *n* = 13 D15), Con mAb + subSNP (*n* = 22 D-7, 1, 21, *n* = 14 D15), CGRP mAb + PBS (*n* = 24 D-7, 1, 21, *n* = 16 D15), CGRP mAb + subSNP (*n* = 24 D-7, 1, 21, *n* = 16 D15). Scatter plot representation of the individual mice at day 15. The mean ± SEM 50% thresholds are presented in all graphs. In scatter plot graph males are represented by open and females by closed symbols. For all panels **p < 0.01, ***p < 0.001, ****p < 0.0001 indicates significance comparing CGRP or SNP with vehicle groups treated with same mAb, ^*p* < 0.05, ^^*p* < 0.01 indicates significance comparing two CGRP groups (treated with different mAb). Statistics are described in Table 1

We then tested the efficacy of the anti-CGRP mAb when administered 1 day prior to the sub-threshold CGRP or SNP trigger. Figure 5A (left panel) shows that compared to administration of an isotype control mAb, the anti-CGRP mAb significantly prevented the decrease in periorbital 50% thresholds observed after sub-threshold CGRP administration at 24 h after antibody administration (D14). At 7 days later (D21) there was only a partial rescue since the anti-CGRP mAb treated mice were not significantly different from control mAb groups given either PBS vehicle or sub-threshold CGRP. Individual mouse data at day 14 disaggregated by sex (open symbols for males, full symbols for females) are shown in Fig. 5A (right panel), with no apparent differences between sexes.

Whether the anti-CGRP mAb could alleviate periorbital hypersensitivity induced by a sub-threshold SNP trigger was then tested (Fig. 5B). Sub-threshold SNP was tested one day after the sub-threshold CGRP treatments. Similar to the CGRP trigger, anti-CGRP mAb has prevented the decrease in periorbital 50% threshold induced by SNP two days after administration (D15) (Fig. 5B, left panel). As with CGRP sensitivity, at 9 days (D22) there was only a partial rescue from SNP treatment since the anti-CGRP mAb treated mice were not significantly different from control mAb groups given either PBS vehicle or sub-threshold SNP. Individual mouse data at day 15 disaggregated by sex (open symbols for males, full symbols for females) are shown in Fig. 5B (right panel), with no apparent differences between sexes.

## Discussion

In this study we showed that timing of anti-CGRP mAb administration determines the therapeutic efficacy of the mAb. Administration immediately before or after repeated closed head injuries was only able to partially reduce persistent hypersensitivity to tactile stimuli, and at best caused a slightly faster recovery of the acute sensitivity. In contrast, administration of the mAb long after the head injuries, but just prior to a sub-threshold trigger, was able to fully prevent hypersensitivity. The optimal timing and efficacy of repeated mAb treatments will need to be confirmed in future experiments, but overall, the current results suggest a role for CGRP in tactile hypersensitivity following mTBI.

Multiple studies have suggested that CGRP may be involved in the pathophysiology of PTH. Counterintuitively, decreased serum CGRP levels have been reported in mTBI patients compared to controls [10, 22] and in soldiers with mTBI who had headache at the time of blood draw compared to those without headache [23]. However, the variability of assays used to measure plasma CGRP levels may be a contributing factor to the uncertainty [24]. Likewise, some preclinical rodent studies have reported lowered CGRP after severe TBI, which correlated with increased mortality [25, 26], and that CGRP administration restored the TBI brain lesion [25]. However, the Pradhan group reported increased CGRP in mouse trigeminal ganglia post mTBI [27] and a recent clinical study reported elevated serum CGRP levels in patients with persistent post-traumatic headache [28]. Importantly, infusion of CGRP caused a higher frequency of migraine-like headaches in patients who had suffered mTBI as compared to placebo [9], which suggests heightened sensitivity to CGRP, as we observed in the mice after mTBI. TBI clinical trials investigating the efficacy of CGRP targeting drugs such as anti-CGRP mAbs are ongoing but preliminary results are conflicting. A single-arm clinical trial investigating the efficacy, tolerability, and safety of the anti-CGRP receptor mAb erenumab for preventive treatment of persistent PTH reported a lower frequency of moderate to severe headache days over the 12-week study [11]. Another case series of five women receiving erenumab for the treatment of PTH reported a 51% reduction in headache intensity compared to before treatment [12]. These reports are consistent with reportedly improved headache severity and frequency in a retrospective analysis of PTH patients who received anti-CGRP mAbs fremanezumab, galcanezumab, or anti-CGRP receptor mAb erenumab [13]. In contradiction with those positive results, a phase 2 study that evaluated the efficacy and safety of the anti-CGRP mAb fremanezumab for the treatment of PTH reported no reduction in headache days compared to placebo [29, 30]. However, a confounder is that there was a fairly high dropout rates of 37% and 80% in the 12 week double blind and subsequent 12 week open label periods, respectively. Thus, given these contradictions, the efficacy of anti-CGRP mAbs in PTH patients remains an open question [13], which may be resolved by results from unreported (NCT03791515, NCT03974360) and on-going (NCT05049057, NCT04098250) clinical trials.

The repetitive closed head injury mTBI protocol we used in this study relies on a single injury modality. It is based on the protocol described by the Levy lab [31]. Similar to the acute and persistent hypersensitivity phases they observed with a nitroglycerin trigger, we also observed acute and persistent hypersensitivity phases in this study and previously [14]. Persistent sensitization to nitroglycerin and bright light stress has also been reported in rodents by the Pradhan and Porreca labs following mTBI [27, 32]. Using SNP as a nitric oxide donor, we also saw persistent hypersensitivity, which could be alleviated by an anti-CGRP mAb. The rescue was similar to that following a CGRP trigger, with no apparent differences between sexes.

The connection between CGRP and nitric oxide is complex. In patients, a small clinical study found that blocking CGRP actions with olcegepant was not able to prevent nitroglycerin-induced migraine [33]. However, a number of preclinical studies indicate that nitric oxide responses are dependent on CGRP action [16, 34]. An interdependence is further supported by cell culture studies showing that nitric oxide increases CGRP synthesis and secretion [35]. Importantly, Geppetti and colleagues reported periorbital sensitization via CGRP signaling in glial cells followed by TRPA1 activation through nitric oxide signaling [36, 37], that is mediated by crosstalk between nitric oxide and hydrogen sulfide [38, 39].

With respect to mechanisms underlying sensitization, Levy and colleagues found that mast cell degranulation was required for development of latent (persistent) sensitization but was not required for the acute sensitization phase [40]. The Levy lab also tested the anti-CGRP mAb fremanezumab on TBI-induced tactile hypersensitivity [31]. In male rats, persistent cephalic tactile pain hypersensitivity to nitroglycerin was reduced by early and prolonged anti-CGRP mAb treatment [31, 41]. Unexpectedly, they found female rats had more robust persistent cephalic tactile sensitivity to nitroglycerin than males after mTBI and that females had a poorer response to early and prolonged anti-CGRP treatment [42]. In contrast, while not fully powered for sex, our data indicate that a similar degree of hypersensitivity in both sexes and that female mice had a greater anti-CGRP mAb response than males. Differences in the experimental paradigms preclude direct comparisons between our data and those of the Levy and Porreca labs (discussed below). Namely, we used a single injection of the humanized anti-CGRP mAb, while they used a mouse version of fremanezumab with early and repeated injections. Future studies will be needed to sort out this apparent sex difference, but overall, the results are consistent with our observation that an anti-CGRP mAb can prevent hypersensitivity. Of note, in a recent paper, the Porreca lab reported a greater efficacy of repeated administration of a CGRP receptor antagonist in female compared to male mice after mTBI, in both the acute and persistent phases of tactile and thermal hypersensitivity [43], once again highlighting a sexual dimorphism of PTH in response to CGRP blocking drugs.

In a different weight drop mTBI protocol, the Porreca group showed that early and repeated CGRP antibody treatment alleviated transient and persistent hypersensitivity (both cephalic and extracephalic) in male mice (they did not test females) [32]. Treatment with anti-CGRP mAb fremanezumab, starting 2 h after mTBI and repeated two more times in 14 days reduced the periorbital and plantar tactile sensitivity to levels comparable to those of sham mice, both immediately after mTBI and after stress-induced allodynia-like responses. However, when a single injection of anti-CGRP mAb was given later (day 10) after mTBI, it failed to prevent a stress-induced cutaneous allodynia-like response. When anti-CGRP mAb was given at 2 h post mTBI and day 7 but not at day 14, it prevented the mTBI-induced allodynia-like behavior, but only partial reduction was seen in stress-induce allodynia-like responses [32]. In addition, anti-CGRP mAb administered shortly after mTBI induction blocked development of cutaneous allodynia-like behavior and prevented the loss of diffuse noxious inhibitory control responses in mice. These data suggest the mTBI-induced PTH-like pain behavior and dysregulation of central pain modulatory mechanisms are mediated by CGRP [44].

There are several limitations to our study. Importantly, we do not know the sites of action or cellular mechanisms by which the anti-CGRP mAb is working to ameliorate tactile hypersensitivity. It seems most likely that CGRP acts at multiple sites and by multiple mechanisms involving actions on immune cells, glia, and neurons in the meninges and the trigeminal ganglia [45]. In the larger picture, tactile hypersensitivity is only a surrogate assessment of a PTH symptom and does not necessarily reflect headache. Nonetheless, allodynia is experienced by 60% of TBI patients in both periorbital and extracephalic areas [46]. An additional limitation is that we did not measure endogenous CGRP levels following TBI or treatments. Future studies are needed to identify these unanswered mechanisms.

In summary, administration of an anti-CGRP mAb can at least partially attenuate mTBI-induced tactile sensitivity. In particular, the ability of the anti-CGRP mAb to substantially prevent the action of both CGRP and SNP triggers when given 1 day prior to the trigger is encouraging. Based on results from our and other laboratories, we hypothesize that repeated treatments with anti-CGRP mAbs may be effective for reducing persistent sensitivities following TBI.

## Supplementary Information

Supplementary Material 1.

Supplementary Material 2: Figure 1. Data from figure 3 separated by sex. A) Scatter plot representation of the individual female and male mice at day 28. B) Scatter plot representation of the individual female and male mice at day 80. For all panels *p < 0.05, **p < 0.01, ***p < 0.001, ****p < 0.0001 indicates significance comparing CGRP and vehicle groups treated with same mAb, ^*p* < 0.05, ^^^^*p* < 0.0001 indicates significance comparing two CGRP groups. Statistics are described in Supplementary Table 1.

Supplementary Material 3: Figure 2. Data from figure 4 separated by sex. Scatter plot representation of the individual female and male mice at day 3. **p < 0.01. Statistics are described in Supplementary Table 1.

## Data Availability

No datasets were generated or analysed during the current study.

## Abbreviations

CGRP: Calcitonin gene-related peptide
mTBI: Mild traumatic brain injury
mAb: Monoclonal antibody
PTH: Post-traumatic headache
SEM: Standard error of the mean
SNP: Sodium nitroprusside
