# Supplementary material for: Pretreatment with an anti-CGRP monoclonal antibody attenuates mild TBI-induced tactile hypersensitivity in mice
Source: J Headache Pain. 2025 Aug 4;26(1):175. doi: 10.1186/s10194-025-02108-x (PMC12323213; doi:10.1186/s10194-025-02108-x)
Supplement: Supplementary file 1 — Supplementary Material 1. [file 10194_2025_2108_MOESM1_ESM.docx]

**Supplementary Table 1.**

| Figure # | Analysis | Statistics (symbol on Figure) |
| --- | --- | --- |
| Supplement Figure 1A  Left panel | Two-way ANOVA  Interaction factor  Treatment factor  Antibody factor  Tukey’s multiple comparison  - Con mAb+PBS vs. Con mAb+CGRP  - Con mAb+PBS vs. CGRP mAb+PBS  - Con mAb+PBS vs. CGRP mAb+CGRP  - Con mAb+CGRP vs. CGRP mAb+PBS  - Con mAb+CGRP vs. CGRP mAb+CGRP  - CGRP mAb+PBS vs. CGRP mAb+CGRP | *F*_(1,31)_=2.631, *p*=0.1149  *F*_(1,31)_=28.71, *p<*0.0001  *F*_(1,31)_=7.109, *p=*0.0121  *P=*0.0001 (***)  *p=*0.8760  *p=*0.2592  *p<*0.0001  *p=*0.0266 (^)  *p=*0.0639 |
| Supplement Figure 1A  Right panel | Two-way ANOVA  Interaction factor  Treatment factor  Antibody factor  Tukey’s multiple comparison  - Con mAb+PBS vs. Con mAb+CGRP  - Con mAb+PBS vs. CGRP mAb+PBS  - Con mAb+PBS vs. CGRP mAb+CGRP  - Con mAb+CGRP vs. CGRP mAb+PBS  - Con mAb+CGRP vs. CGRP mAb+CGRP  - CGRP mAb+PBS vs. CGRP mAb+CGRP | *F*_(1,32)_=0.7735, *p*=0.3857  *F*_(1,32)_=24.38, *p<*0.0001  *F*_(1,32)_=2.744, *p=*0.1074  *P=*0.0014 (**)  *p=*0.9460  *p=*0.1146  *p=*0.0003  *p=*0.2951  *p=*0.0346 (*) |
| Supplement Figure 1B  Left panel | Two-way ANOVA  Interaction factor  Treatment factor  Antibody factor  Tukey’s multiple comparison  - Con mAb+PBS vs. Con mAb+CGRP  - Con mAb+PBS vs. CGRP mAb+PBS  - Con mAb+PBS vs. CGRP mAb+CGRP  - Con mAb+CGRP vs. CGRP mAb+PBS  - Con mAb+CGRP vs. CGRP mAb+CGRP  - CGRP mAb+PBS vs. CGRP mAb+CGRP | *F*_(1,31)_=14.82, *p*=0.0006  *F*_(1,31)_=42.37, *p<*0.0001  *F*_(1,31)_=11.83, *p=*0.0017  *p<*0.0001 (****)  *p=*0.9909  *p=*0.1635  *p<*0.0001  *p<*0.0001 (^^^^)  *p=*0.2690 |
| Supplement Figure 1B  Right panel | Two-way ANOVA  Interaction factor  Treatment factor  Antibody factor  Tukey’s multiple comparison  - Con mAb+PBS vs. Con mAb+CGRP  - Con mAb+PBS vs. CGRP mAb+PBS  - Con mAb+PBS vs. CGRP mAb+CGRP  - Con mAb+CGRP vs. CGRP mAb+PBS  - Con mAb+CGRP vs. CGRP mAb+CGRP  - CGRP mAb+PBS vs. CGRP mAb+CGRP | *F*_(1,32)_=0.5525, *p*=0.4627  *F*_(1,32)_=10.78, *p=*0.0025  *F*_(1,32)_=0.4243, *p=*0.5194  *P=*0.0364 (*)  *p=*0.9999  *p=*0.2645  *p=*0.0423  *p=*0.7582  *p=*0.2936 |
| Supplement Figure 2, Left | t test | *p*=0.0042 (**) |
| Supplement Figure 2, Right | t test | *p*=0.5769 |
