# Supplementary figures and images for: Pretreatment with an anti-CGRP monoclonal antibody attenuates mild TBI-induced tactile hypersensitivity in mice
Source: J Headache Pain. 2025 Aug 4;26(1):175. doi: 10.1186/s10194-025-02108-x (PMC12323213; doi:10.1186/s10194-025-02108-x)

**A**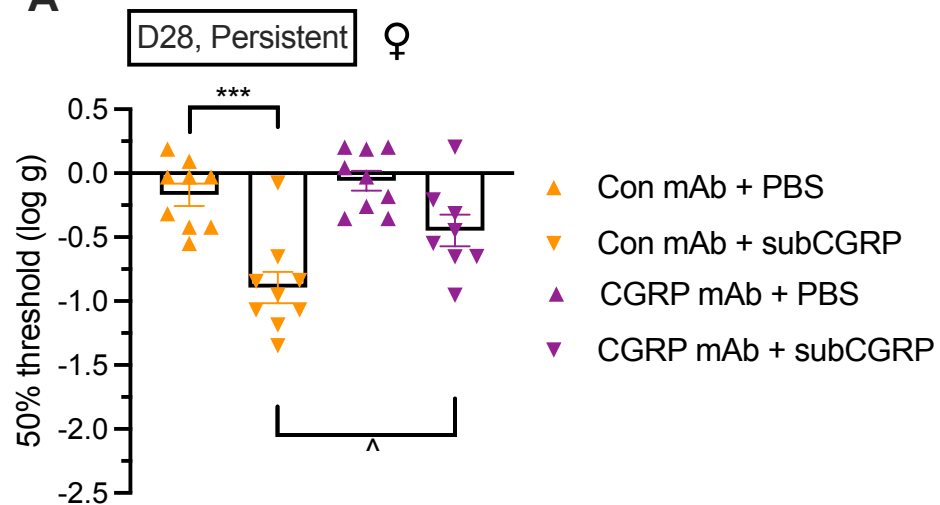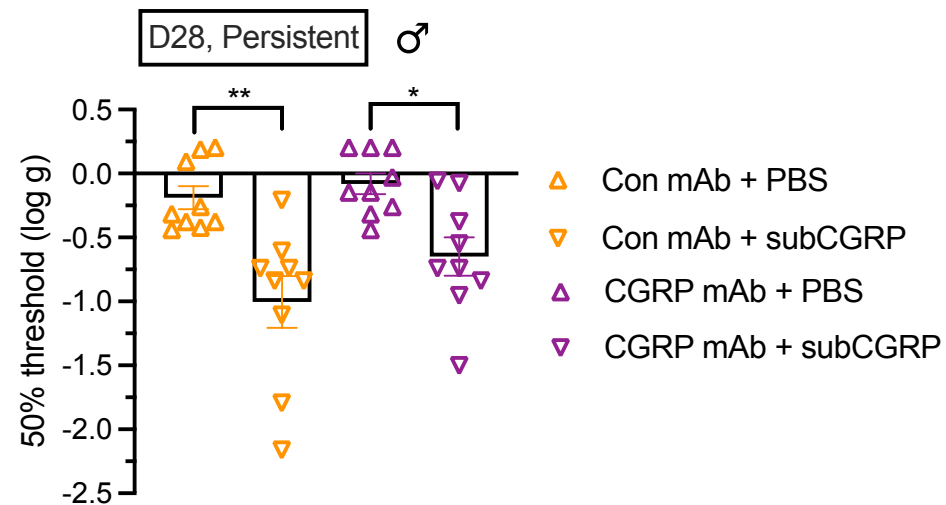**B**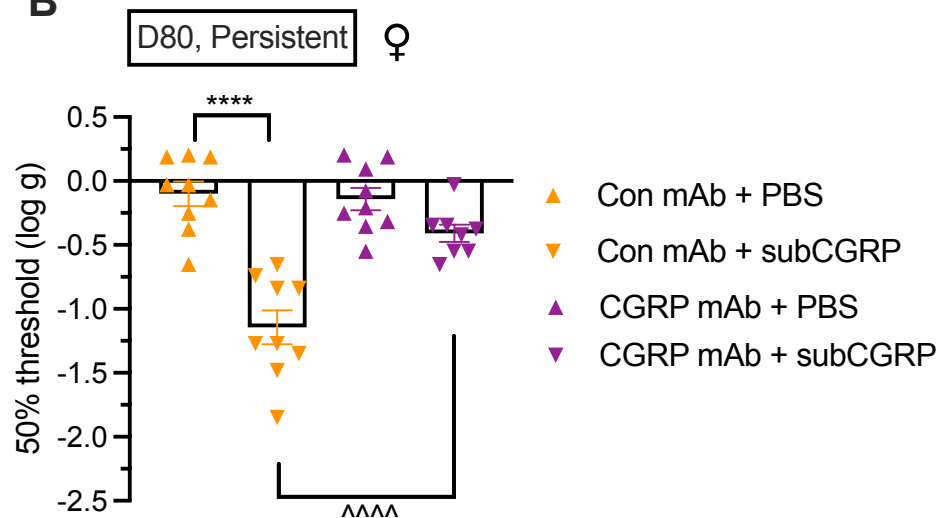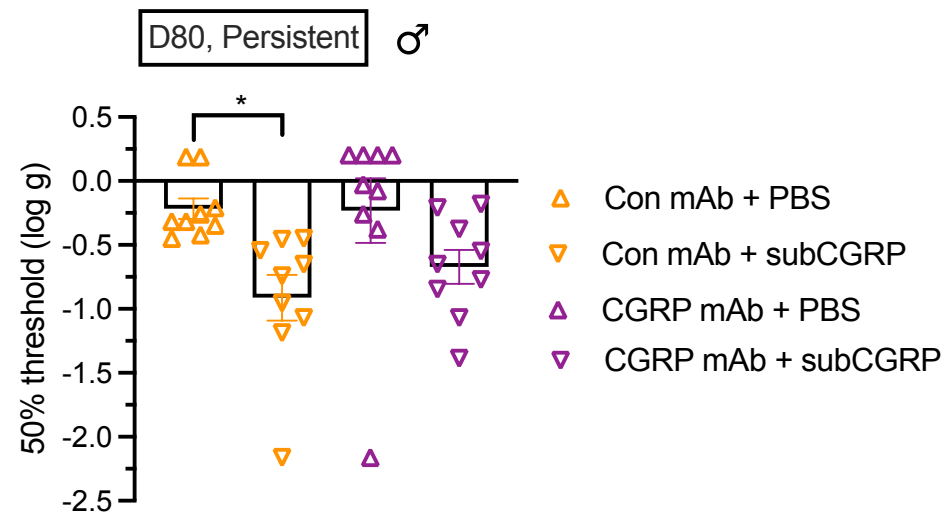

Supplement: Supplementary file 2 — Supplementary Material 2: Figure 1. Data from figure 3 separated by sex. A) Scatter plot representation of the individual female and male mice at day 28. B) Scatter plot representation of the individual female and male mice at day 80. For all panels *p < 0.05, **p < 0.01, ***p < 0.001, ****p < 0.0001 indicates significance comparing CGRP and vehicle groups treated with same mAb, ^p < 0.05, ^^^^p < 0.0001 indicates significance comparing two CGRP groups. Statistics are described in Supplementary Table 1. [file 10194_2025_2108_MOESM2_ESM.pdf]

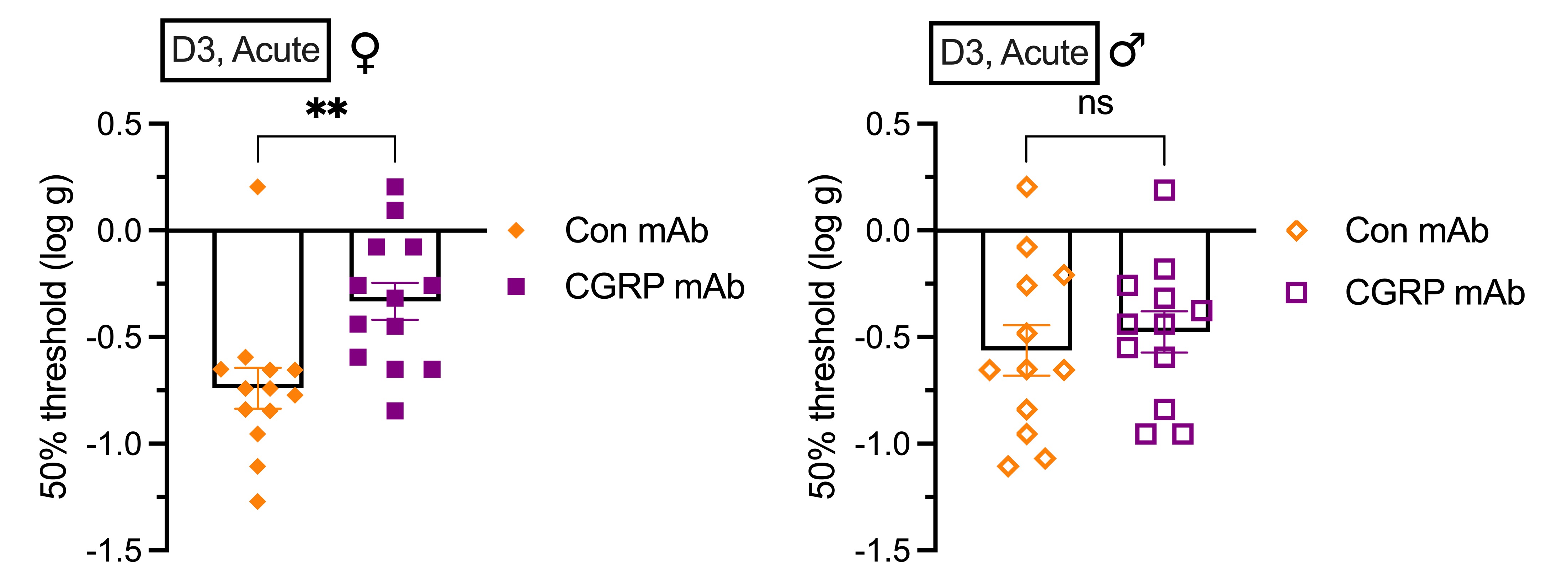

Supplement: Supplementary file 3 — Supplementary Material 3: Figure 2. Data from figure 4 separated by sex. Scatter plot representation of the individual female and male mice at day 3. **p < 0.01. Statistics are described in Supplementary Table 1. [file 10194_2025_2108_MOESM3_ESM.jpg]
